# Association of Hepatitis B and Hepatitis C Virus Infections With Hepatocellular Carcinoma: A Systematic Review of Virological, Histopathological, and Molecular Evidence

**DOI:** 10.7759/cureus.117388

**Published:** 2026-10-03

**Authors:** Ritu Saxena, Smriti Varshney, Mohd Sanan Khan, Mohit Bansal, Ruksar Syed, Patel Keta Sanjaybhai, Kuldeep Singh, Rajdeep Paul

**Affiliations:** 1 Pathology, Shri Siddhi Vinayak Medical College, Sambhal, IND; 2 Family Medicine, Aashirwad Healthcare Centre, Chandausi, IND; 3 Pediatrics, University College of Medical Sciences and Guru Teg Bahadur (GTB) Hospital, Delhi, IND; 4 Pathology, B.J. Medical College, Ahmedabad, IND; 5 Clinical Microbiology, Mahaveer Institute of Medical Sciences and Research, Bhopal, IND

**Keywords:** cirrhosis, epigenetics, hbv integration, hcv rna, hepatitis b virus, hepatitis c virus, hepatocellular carcinoma, histopathology, molecular carcinogenesis, viral load

## Abstract

Chronic hepatitis B virus (HBV) and hepatitis C virus (HCV) infections are major but biologically distinct causes of hepatocellular carcinoma (HCC). This systematic review synthesized virological, histopathological, genomic, and molecular evidence linking HBV and HCV infections with HCC and compared their dominant pathways of hepatocarcinogenesis. The review was conducted in accordance with the Preferred Reporting Items for Systematic Reviews and Meta-Analyses 2020 guidelines. PubMed/MEDLINE was searched from inception through June 30, 2026, supplemented by backward citation searching. Human primary studies evaluating HBV- or HCV-associated HCC using virological, histopathological, genomic, epigenetic, or molecular outcomes were eligible. Of 310 records identified, 278 were screened after duplicate removal, 60 full-text reports were assessed, and 25 studies were included in the qualitative synthesis. Meta-analysis was not undertaken because of substantial clinical and methodological heterogeneity.

Prospective HBV studies demonstrated strong virological risk gradients. Combined hepatitis B surface antigen and hepatitis B e antigen positivity was associated with an adjusted relative risk of 60.2 (95% confidence interval: 35.5-102.1) for HCC, while HCC incidence increased from 108 to 1,152 per 100,000 person-years across increasing HBV DNA categories. Molecular studies consistently demonstrated enrichment of HBV DNA integration in tumor tissue, with recurrent involvement of telomerase reverse transcriptase and other cancer-associated loci, together with insertional mutagenesis and structural genomic alterations. In HCV infection, cumulative HCC risk increased from 1.1% among HCV RNA-negative individuals to 6.4% and 14.7% among low- and high-viremia groups, respectively. HCV-associated HCC was more closely linked to chronic necroinflammation, oxidative injury, fibrosis, cirrhosis, viral protein-mediated signaling, and persistent epigenetic alterations rather than viral genome integration. Histopathological evidence generally demonstrated greater background necroinflammation and cirrhosis in HCV-associated HCC, whereas HBV-associated HCC could occur in less fibrotic or noncirrhotic liver.

HBV and HCV therefore converge on hepatocarcinogenesis through chronic liver injury but differ in their dominant mechanisms. HBV has a prominent direct oncogenic component involving viral DNA integration and genomic instability, whereas HCV-associated HCC is predominantly driven by inflammation-, fibrosis-, and cirrhosis-associated carcinogenesis with additional molecular and epigenetic effects. Viral suppression or eradication reduces but does not completely eliminate HCC risk after advanced hepatic or persistent molecular injury has developed.

## Introduction and background

Hepatocellular carcinoma (HCC) is the predominant primary malignancy of the liver and accounts for most primary liver cancer cases. Its development generally reflects a multistep process involving chronic hepatocyte injury, compensatory regeneration, fibrosis, genomic damage, and clonal selection. Chronic hepatitis B virus (HBV) and hepatitis C virus (HCV) infection remain major global etiologic factors for HCC, although their contributions vary geographically and their dominant pathways of malignant transformation differ [1,2].

HBV shows a strong epidemiological association with HCC. In a prospective cohort of 11,893 Taiwanese men, Yang et al. reported a marked increase in HCC risk among individuals with markers of active HBV replication. Participants positive for both hepatitis B surface antigen (HBsAg) and hepatitis B e antigen (HBeAg) had an adjusted relative risk of 60.2 compared with individuals negative for both markers [3]. In a later cohort of 3,653 HBsAg-positive participants, Chen et al. demonstrated a clear dose-response relationship between serum HBV DNA and HCC risk, with incidence increasing from 108 to 1,152 per 100,000 person-years across viral-load categories [4].

HBV-related risk is also influenced by viral genetic variation. The T1762/A1764 basal core promoter mutation has been associated with a substantially higher risk of HCC [5]. Longitudinal data indicate that enhancer II/basal core promoter variants may be detectable years before tumor development [6], and increasing numbers of high-risk mutations involving T1653, V1753, and T1762/A1764 have been linked to progressively greater HCC risk [7]. More recent cohort data suggest that HBV DNA level remains an important determinant of HCC risk even in noncirrhotic chronic hepatitis B [8].

A notable feature of HBV-associated carcinogenesis is that it is not explained by inflammation and cirrhosis alone. HBV DNA can integrate into the host genome, creating a direct route to genomic disruption. Genome-wide studies have shown recurrent HBV integration in HCC tissue, including events involving cancer-related loci such as telomerase reverse transcriptase (TERT), KMT2B/MLL4, and CCNE1 [9]. Genomic and transcriptomic analyses have also identified integration-associated gene disruption, viral-host fusion transcripts, copy-number changes, and clonal expansion [10]. Deep-sequencing studies demonstrated recurrent TERT involvement and structural alterations involving the HBx region [11], while newer whole-genome and long-read sequencing analyses have revealed integration-associated rearrangements and oncogenic regulatory effects [12,13]. These observations support a direct contribution of HBV to hepatocarcinogenesis and help explain why HCC can arise before cirrhosis in some patients.

HCV-associated carcinogenesis differs in important respects. HCV is an RNA virus and does not normally integrate into host chromosomal DNA. Its association with HCC is therefore more closely linked to persistent infection, chronic inflammation, oxidative injury, repeated hepatocyte turnover, fibrosis, and cirrhosis. In a population-based cohort of 925 anti-HCV-positive individuals, cumulative HCC risk was 1.1%, 6.4%, and 14.7% in nonviremic, low-viremic, and high-viremic groups, respectively [14]. Viral genotype may also influence risk: genotype 1b was more frequent in HCC cases than cirrhotic controls in a case-control study [15], and genotype 1b was present in 19 of 22 incident HCC cases in a prospective cirrhosis cohort [16].

Molecular effects of HCV may provide additional steps toward malignant transformation. HCV core-gene variation has been linked to advanced liver disease and HCC, and greater sequence variability has been observed in tumor compared with nontumor tissue [17,18]. These findings suggest that direct molecular effects of HCV may contribute alongside the dominant roles of chronic inflammation and fibrosis.

Histopathological comparisons support these biological differences. In noncirrhotic HCC, HCV-associated background liver showed greater necroinflammatory activity than HBV-associated liver despite similar fibrosis [19]. Other clinicopathological studies reported older age and more frequent cirrhosis in HCV-associated HCC, whereas HBV-associated tumors tended to be larger and less differentiated [20]. Greater synchronous multicentricity has also been reported in HCV-related HCC, consistent with field carcinogenesis in chronically injured liver [21].

The carcinogenic effect of chronic viral hepatitis may persist after virological control. HCV clearance has been associated with persistent enhancer and chromatin changes, supporting the concept of an epigenetic scar [22]. Residual HCC risk after successful HCV eradication has also been reported, suggesting that accumulated molecular and structural liver injury may not be fully reversed [23].

Mechanistic reviews have emphasized both direct and indirect pathways in HBV-related carcinogenesis and the major roles of chronic inflammatory, oxidative, and fibrotic injury in HCV-related HCC [24,25]. However, virological markers, histopathological changes, and molecular findings are often considered separately. Integrating these evidence streams may better distinguish the dominant mechanisms of HBV and HCV carcinogenesis and clarify clinically relevant implications for surveillance after viral suppression or eradication.

The aim of this systematic review was therefore to summarize available virological, histopathological, genomic, and molecular evidence concerning the association of HBV and HCV infections with HCC, with particular attention to HBV serological markers, viral load, high-risk mutations, and viral-host integration; HCV RNA and genotype; comparative histopathology; HBV/HCV coinfection and occult HBV infection; and persistent molecular or epigenetic alterations associated with hepatocarcinogenesis.

Rationale

This review was designed to integrate three evidence streams that are commonly reported separately: prospective virological risk associations, histopathological differences between tumor and background liver, and molecular mechanisms such as HBV integration, viral mutations, and HCV-associated epigenetic reprogramming. The integrated approach was intended to distinguish epidemiological association from biological mechanism and to relate both to clinically relevant HCC surveillance after viral suppression or eradication.

Review question and objectives

The review asked which virological, histopathological, and molecular findings support an association between current or previous HBV and/or HCV infection and HCC, and how the dominant HCC-promoting pathways differ between the two viruses. Accordingly, the objectives were to evaluate virological predictors of HBV-associated HCC, including HBsAg, HBeAg, HBV DNA, and viral mutations; evaluate virological predictors of HCV-associated HCC, including HCV RNA and viral genotype; compare the histopathological background and tumor characteristics of HBV- and HCV-associated HCC; synthesize evidence on HBV integration, HBx-related effects, and host-genome disruption; synthesize HCV molecular evidence, including core-gene variation and persistent epigenetic alterations; evaluate evidence relating HBV/HCV coinfection and occult HBV infection to HCC; and assess methodological quality, reporting bias, and certainty of the evidence.

## Review

Materials and methods

Study Design and Reporting

This systematic review evaluated virological, histopathological, genomic, and molecular evidence linking HBV and HCV infection with HCC and was prepared in accordance with the Preferred Reporting Items for Systematic Reviews and Meta-Analyses (PRISMA) 2020 statement [26]. Because the eligible literature included prospective cohorts, case-control studies, clinicopathological series, genomic sequencing studies, and molecular investigations with substantially different outcomes and effect measures, a quantitative meta-analysis was not planned.

This was a multi-institutional collaborative review involving authors from pathology, family medicine, pediatrics, and clinical microbiology affiliations. The collaboration involved review design, literature appraisal, interpretation of the evidence, manuscript drafting, and critical revision. Because the study synthesized previously published literature, it involved no direct patient recruitment or laboratory experimentation. Individual author roles are specified in the Author Contributions section.

Eligibility Criteria

Eligibility criteria were structured using the Population-Exposure-Outcome-Study Design (PEOS) framework, a framework used to formulate focused questions and eligibility criteria for reviews of exposure-related or observational evidence [27]. The population comprised human participants with current or previous HBV and/or HCV infection, as well as human HCC tumor or adjacent nontumor tissue in which HBV or HCV status was clearly characterized. HBV-related exposures included HBsAg, HBeAg, HBV DNA, viral genotype or mutation, HBV DNA integration, and other viral-genome characteristics. HCV-related exposures included HCV RNA, HCV genotype, viral sequence variation, and molecular or epigenetic alterations; studies of HBV/HCV coinfection and occult HBV infection were also eligible. Outcomes included incident HCC, HCC risk, tumor or background-liver histopathology, viral-host integration, structural genomic abnormalities, viral-sequence changes, epigenetic abnormalities, and other molecular features directly relevant to hepatocarcinogenesis. Eligible study designs included prospective and retrospective cohort studies, case-control studies, comparative histopathological studies, molecular or genomic tissue studies, and other primary human investigations available in full text through June 30, 2026.

Exclusion Criteria

Studies were excluded when they were reviews, systematic reviews, meta-analyses, editorials, commentaries, or letters without extractable primary data; did not report an HCC outcome or did not clearly define HBV or HCV exposure; were animal or in vitro studies without directly extractable human HCC data; examined liver tumors other than HCC without separable HCC-specific findings; lacked extractable virological, histopathological, genomic, epigenetic, or molecular outcomes; represented duplicate or substantially overlapping study populations without additional relevant information; or provided insufficient information for meaningful interpretation.

Information Sources and Search Period

PubMed/MEDLINE was searched through the PubMed interface from database inception to June 30, 2026. Reference lists of included studies and highly relevant background articles were also examined manually to identify additional eligible reports. Trial registries and regulatory databases were not searched because the review addressed etiological, pathological, and molecular evidence rather than therapeutic efficacy. Study authors and manufacturers were not contacted for additional records or unpublished data.

Search Strategy

The PubMed search combined controlled vocabulary and free-text terms for hepatocellular carcinoma, HBV/HCV infection, and virological, histopathological, genomic, and molecular mechanisms. No automatic article-type filter was applied because study eligibility was determined during screening. The complete search strategy, including the final Boolean string and search date, is provided in the Appendix.

No machine-learning classifier, natural-language processing tool, automated translation system, or automated screening platform was used.

Study Selection

All retrieved records were screened against the predefined eligibility criteria. Title and abstract screening was followed by full-text assessment of potentially relevant reports. Screening was performed by one reviewer and was not independently duplicated. When eligibility was uncertain, the full article was reassessed against the predefined PEOS criteria before a final decision was made. No crowdsourcing or automated eligibility assessment was used, and study investigators were not contacted to resolve uncertain eligibility.

Data Extraction

Data were extracted manually into a structured evidence table. For each included study, the extraction captured the first author and publication year; country or study setting; study design and sample size; HBV or HCV status; serological markers, viral load, genotype, or mutations; fibrosis, cirrhosis, and necroinflammation; tumor and background-liver histopathology; HBV integration frequency and targets; viral-host fusion events and structural genomic abnormalities; HCV sequence variation and molecular features; epigenetic alterations; incident HCC or HCC risk estimates; reported relative risks, hazard ratios (HRs), odds ratios (ORs), incidence rates, or cumulative incidence; follow-up duration; and major methodological limitations. Numerical values were retained as reported in the original studies, missing values were recorded as not reported rather than estimated or imputed, and where reports appeared to involve overlapping populations, the report containing the most complete or relevant outcome data was prioritized.

Risk-of-Bias and Methodological Quality Assessment

Risk of bias was assessed by one reviewer. Epidemiological cohort, case-control, and analytical observational studies were evaluated using design-specific domains derived from the Joanna Briggs Institute critical appraisal framework for etiology and risk studies [28]. Molecular and genomic studies were additionally assessed using domains informed by the Quality of Genetic Studies (Q-Genie) tool [29]. Because several sequencing and tumor-genomic studies were not conventional genetic-association studies, Q-Genie domains were adapted to evaluate sample selection, assay or sequencing validity, technical error, confounding, statistical analysis, and biological interpretation rather than applying a numerical Q-Genie score. Overall judgments were categorized as low concern, some concern, or great concern and reflected the most important limitations related to sample selection, exposure or assay validity, comparator groups, confounding, outcome validity, completeness of data, and analysis.

Effect Measures

For etiological studies, effect estimates were extracted as reported in the original publications, including relative risks, HRs, ORs, incidence rates, cumulative incidence, and 95% confidence intervals (CIs). For molecular, genomic, and histopathological studies, proportions, frequencies, sequence findings, integration events, and comparative descriptive results were retained in their original form. Effect measures were not converted to a common scale because of substantial differences in study design and outcome definition.

Data Synthesis

Studies were synthesized narratively across seven prespecified evidence domains: HBV serological markers and viral load; HBV viral mutations; HCV RNA and genotype; HBV/HCV coinfection and occult HBV infection; comparative histopathological findings; HBV DNA integration and genomic alterations; and HCV molecular and epigenetic evidence, including residual HCC risk after viral eradication. Narrative synthesis was selected because the included studies differed substantially in population characteristics, viral exposures, laboratory techniques, sequencing platforms, histopathological measurements, duration of follow-up, and reported effect measures. The synthesis considered the direction and magnitude of associations, temporal relationships, biological gradients, replication across independent studies, and consistency between epidemiological and molecular evidence.

Assessment of Heterogeneity

Clinical and methodological heterogeneity was assessed qualitatively according to viral infection, geographic region, cirrhosis status, study design, viral assay, sequencing or molecular technique, tissue source, and treatment era. Because the evidence was not sufficiently homogeneous, statistical subgroup analysis, meta-regression, and pooled measures of heterogeneity were not performed.

Sensitivity Analysis

A qualitative sensitivity analysis reassessed the principal conclusions after assigning minimal interpretive weight to studies judged to have high methodological concern. This approach was used to determine whether the main conclusions depended heavily on small or methodologically limited tissue studies.

Reporting-Bias Assessment

Reporting bias was assessed using a structured qualitative adaptation of the Risk Of Bias due to Missing Evidence (ROB-ME) framework [30]. ROB-ME is designed to evaluate bias arising when studies or study results are missing from a synthesis because of the direction, magnitude, or statistical significance of findings. Because this review did not undertake meta-analysis and did not generate a common pooled effect estimate, the full ROB-ME algorithm, funnel plots, and regression-based tests for small-study effects were not applicable. Instead, ROB-ME concepts were used narratively to consider the likelihood of missing studies, selective nonreporting of outcomes, the availability of prospective protocols or analysis plans, and whether susceptibility to reporting bias differed between large prospective cohorts and small exploratory molecular or tissue studies. No numerical ROB-ME score was assigned.

For the qualitative assessment, lower susceptibility to reporting bias was assigned to evidence derived from larger prospective or population-based studies with clearly defined exposures and outcomes, complete reporting of relevant outcomes, systematic follow-up, and available or clearly described prespecified analytical methods. Greater susceptibility was assigned when prospective protocols or analysis plans were unavailable, outcome reporting appeared incomplete or selectively emphasized statistically significant or positive findings, studies were small or exploratory, multiple molecular outcomes were examined without clear prespecification, or unpublished null findings were considered plausible. Evidence domains dominated by small exploratory molecular or tissue-based investigations were therefore considered more susceptible to reporting bias than those supported by large prospective cohorts.

Certainty of Evidence

A GRADE-informed narrative approach was used to summarize certainty of evidence [31]. Because most of the evidence was observational, certainty generally began at a low level and was considered for upgrading when there was a large effect, clear biological gradient, strong temporal relationship, or mechanistic replication. Certainty was downgraded for important concerns related to risk of bias, inconsistency, indirectness, imprecision, or possible reporting bias. Final judgments were categorized as very low, low, moderate, or high certainty.

Registration of Protocol

The review was not prospectively registered, and no publicly accessible protocol was prepared. The eligibility criteria, evidence domains, and narrative-synthesis approach used in the manuscript were established before completion of the final full-text synthesis.

Results

Study Selection

The search identified 310 records, including 286 from PubMed/MEDLINE and 24 through citation searching and other sources. After removal of 32 duplicates, 278 records underwent title and abstract screening. Of these, 215 were excluded, leaving 63 reports for full-text retrieval. The 215 records excluded during title and abstract screening clearly failed the predefined eligibility criteria, principally because they were nonprimary publications, did not evaluate HCC, did not adequately characterize HBV or HCV exposure, were animal or in vitro investigations, or did not report relevant virological, histopathological, genomic, epigenetic, or molecular outcomes. Individual category counts were not recorded at the title and abstract screening stage. Three reports could not be retrieved, and 60 full-text articles were assessed for eligibility. Thirty-five reports were excluded, resulting in 25 primary studies being included in the qualitative synthesis [3-23,32-35]. 

The 35 full-text exclusions comprised reviews, systematic reviews, meta-analyses, or editorials (n = 10); studies without a relevant HCC outcome (n = 6); inadequately characterized HBV/HCV exposure (n = 3); exclusively animal or in vitro studies (n = 5); studies without extractable virological, histopathological, genomic, epigenetic, or molecular findings (n = 7); duplicate or overlapping study populations (n = 2); and other reasons (n = 2). The study-selection process is summarized in Figure 1.

**Figure 1:**
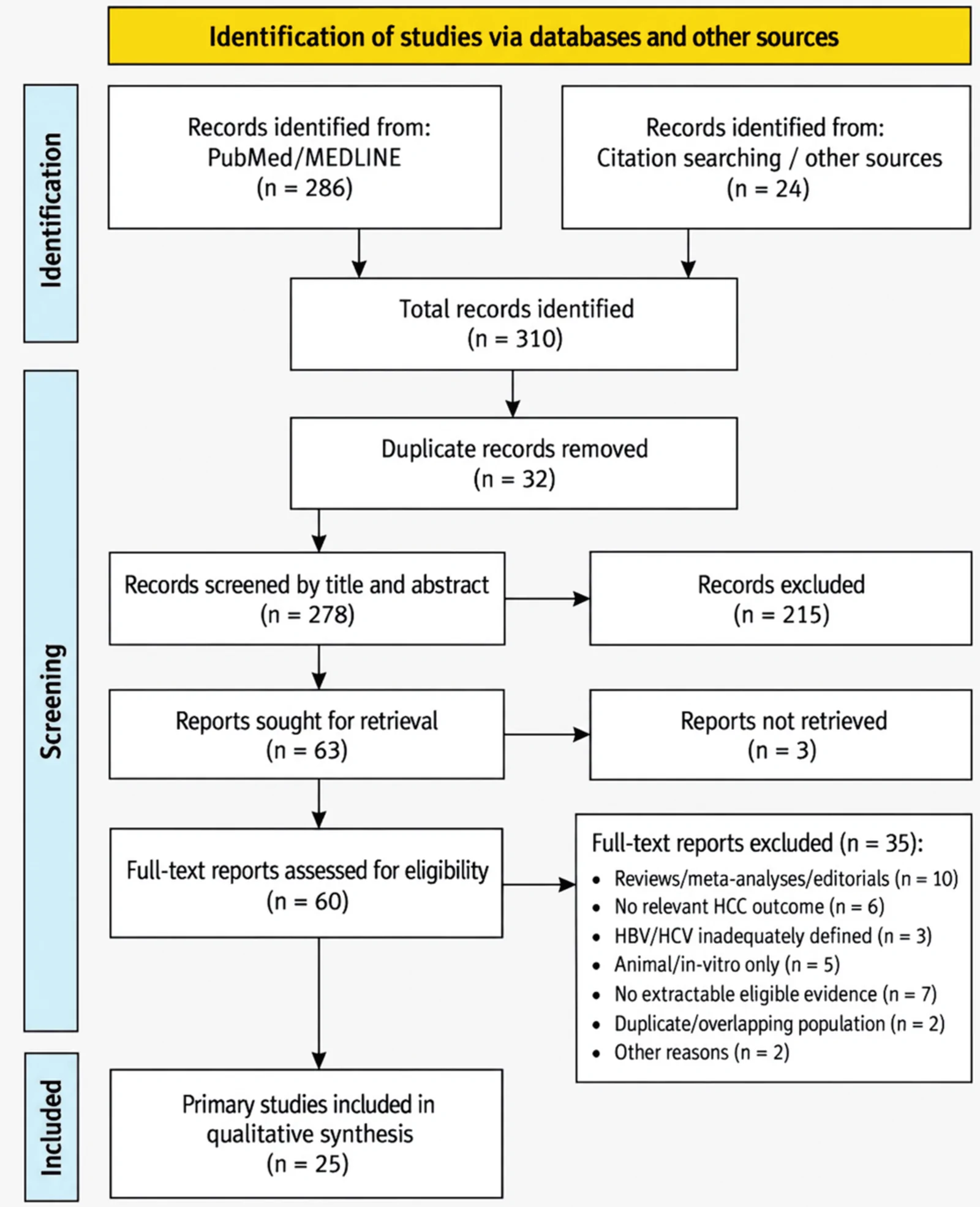
PRISMA 2020 flow diagram of study selection The diagram summarizes record identification, screening, full-text assessment, exclusions, and inclusion of 25 primary studies in the qualitative synthesis. The flow diagram was prepared according to the PRISMA 2020 framework [26] HCC, hepatocellular carcinoma; HBV, hepatitis B virus; HCV, hepatitis C virus; PRISMA, Preferred Reporting Items for Systematic Reviews and Meta-Analyses

Characteristics of Included Studies

The 25 included primary studies comprised prospective population-based cohorts, cohorts of patients with cirrhosis, nested case-control studies, comparative clinicopathological investigations, tumor and nontumor sequencing studies, molecular association studies, epigenomic investigations, and post-antiviral cohorts. Studies were conducted across Taiwan, China, Korea, Japan, Italy, Germany, the United States, and other international settings, with publication dates ranging from the 1990s to 2025. Their characteristics and principal findings are summarized in Table 1.

**Table 1:** Characteristics and principal findings of included primary studies BCP, basal core promoter; CHB, chronic hepatitis B, CI, confidence interval; DAA, direct-acting antiviral; HBsAg, hepatitis B surface antigen; HBeAg, hepatitis B e antigen; HBV, hepatitis B virus; HBx, hepatitis B virus X; HCC, hepatocellular carcinoma; HR, hazard ratio; NS5, nonstructural 5; OR, odds ratio; PY, person-years; RR, relative risk; TERT, telomerase reverse transcriptase; CCNE1, cyclin E1; WGS, whole-genome sequencing

Study	Setting	Design/sample	Virus	Primary domain	Principal result/effect estimate
Yang et al. 2002 [3]	Taiwan	Prospective cohort; n = 11,893 men	HBV	HBeAg/HBsAg	HCC incidence 39 vs. 324 vs. 1,169/100,000 PY; HBsAg+ RR 9.6 (95% CI, 6.0-15.2); HBsAg+/HBeAg+ RR 60.2 (35.5-102.1)
Chen et al. 2006 [4]	Taiwan	Prospective cohort; n = 3,653 HBsAg+	HBV	HBV DNA	HCC incidence increased from 108 to 1,152/100,000 PY; highest baseline DNA category crude HR 10.7 (95% CI, 5.7-20.1) vs. <300 copies/mL
Kao et al. 2003 [5]	Taiwan	HBV carrier comparison; n = 250	HBV	BCP mutation	T1762/A1764 mutation strongly associated with HCC; adjusted OR 10.60
Chou et al 2008 [6]	Taiwan	Nested case-control within 4,841 carriers	HBV	EnhII/BCP variants	High-risk variants preceded HCC diagnosis; persistent variants strengthened the association
Qu et al. 2014 [7]	China	Molecular epidemiological study	HBV	T1653/V1753/T1762-A1764	HCC risk increased with accumulation of high-risk mutations
Kim et al. 2020 [8]	Korea	Cohort; n = 6,949 noncirrhotic CHB	HBV	HBV DNA	Highest adjusted HCC risk at approximately 6-7 log10 IU/mL; adjusted HR approximately 4.98 vs. low-DNA reference
Sung et al. 2012 [9]	Asia	Tumor sequencing; 81 HBV+ HCCs	HBV	Integration	HBV integration in 86.4% of tumors vs. 30.7% adjacent liver; recurrent TERT, KMT2B/MLL4 and CCNE1 events
Jiang et al. 2012 [10]	Multicenter/Asia	Genomic/transcriptomic analysis	HBV	Integration/genomics	255 integration sites; gene disruption, fusion transcripts, copy-number changes and clonal expansion
Toh et al. 2013 [11]	Asia	Deep sequencing; 48 HCCs	HBV	Integration/HBx	Promoter-enriched integration, recurrent TERT involvement and HBx-region structural alterations
Qian et al. 2024 [12]	China	WGS/long-read sequencing; 124 HCCs	HBV	Structural genomics	Integration-associated structural rearrangements/translocations and driver copy-number alterations
Wang et al. 2025 [13]	China	Long-read sequencing	HBV	Integration/early-onset HCC	Integrated HBV concatemers associated with large rearrangements, MYC amplification and cis-regulatory effects
Lee et al. 2010 [14]	Taiwan	Prospective cohort; n = 925 anti-HCV+	HCV	HCV RNA/genotype	Cumulative HCC risk 1.1% RNA-negative, 6.4% low RNA and 14.7% high RNA; genotype 1 risk 12.6% vs. 4.5%
Silini et al. 1996 [15]	Italy	Case-control in cirrhosis	HCV	Genotype	Genotype 1b more frequent among HCC cases than cirrhotic controls without HCC
Bruno et al. 1997 [16]	Italy	Prospective HCV-cirrhosis cohort; n = 163	HCV	Genotype 1b	19/22 incident HCCs had genotype 1b; OR 6.14
Fishman et al. 2009 [17]	USA/international	Molecular association study	HCV	Core-gene variants	Several core-gene polymorphisms associated with advanced liver disease/HCC; prospective validation required
Rüster et al. 2001 [18]	Germany	Tumor vs. adjacent tissue; n = 8	HCV	Core/NS5 sequence	Greater core-region variability in tumor tissue; substitutions, premature stop codons and frameshift deletions observed
Muro et al. 1996 [19]	Japan	Noncirrhotic HCC pathology; HBV n = 10, HCV n = 26	HBV/HCV	Histopathology	Similar fibrosis; greater necroinflammatory activity in HCV-associated background liver
Takazawa et al. 1996 [20]	Japan	Resected cirrhotic HCC; HCV n = 103, HBV n = 34	HBV/HCV	Clinicopathology	HCV patients older and more cirrhotic; HBV tumors tended to be larger and less differentiated
Miyagawa et al. 1996 [21]	Japan	Surgical HCC cohort; n = 175	HBV/HCV	Multicentricity	HCV group older, poorer liver function and greater synchronous multicentricity
Hlady et al. 2022 [22]	USA	Human molecular epigenomic study	HCV	Epigenetic scar	HCV-associated enhancer/chromatin changes persisted after viral clearance and were demonstrated in human samples
Kondili et al. 2023 [23]	Italy	Post-DAA cirrhosis cohort; n = 2,064 SVR	HCV	Residual HCC risk	119 de novo HCCs; cumulative incidence 1.90%, 4.21% and 6.47% at 12, 24 and 36 months
Benvegnù et al. 1994 [32]	Italy	Prospective cirrhosis cohort; n = 290	HBV/HCV	Coinfection	Concurrent HBV/HCV infection associated with the greatest occurrence of HCC
Tsai et al. 1997 [33]	Taiwan	Prospective cirrhosis cohort; n = 400	HBV/HCV	Coinfection	Coinfection HR 6.41 (95% CI, 1.80-22.80); anti-HCV alone HR 3.74; HBsAg alone HR 4.06
Squadrito et al. 2006 [34]	Italy	HBsAg-negative chronic hepatitis cohort	Occult HBV/HCV	Occult HBV	Eight of nine incident HCCs occurred in occult-HBV-positive patients
Ikeda et al. 2007 [35]	Japan	Prospective chronic HCV cohort; n = 872	HCV/past HBV	Anti-HBc/occult-HBV proxy	Anti-HBc positivity associated with greater HCC risk in cirrhotic HCV patients; incidence rate ratio 1.58

HBV Serological Markers and Viral Replication

Yang et al. demonstrated a marked gradient in HCC risk according to HBV replicative status. HBsAg positivity alone was associated with an adjusted relative risk of 9.6 (95% CI, 6.0-15.2), whereas individuals positive for both HBsAg and HBeAg had an adjusted relative risk of 60.2 (95% CI, 35.5-102.1) [3]. Chen et al. demonstrated a dose-response relationship between baseline HBV DNA and subsequent HCC development. Among 3,653 HBsAg-positive adults, HCC incidence increased from 108 per 100,000 person-years in the lowest viral-load category to 1,152 per 100,000 person-years at least one million copies/mL; the crude HR for the highest baseline category was 10.7 (95% CI, 5.7-20.1) compared with less than 300 copies/mL [4]. Kim et al. subsequently reported that, among treatment-naïve patients with noncirrhotic chronic hepatitis B, HCC risk was highest at intermediate-to-high HBV DNA concentrations of approximately 6-7 log10 IU/mL [8].

HBV Viral Mutations

Specific HBV sequence variants were also associated with HCC risk. Kao et al. reported a strong association between the T1762/A1764 basal core promoter double mutation and HCC, with an adjusted OR of 10.60 [5]. Chou et al. showed that high-risk enhancer II/basal core promoter variants could be detected several years before HCC diagnosis and that persistent variants were particularly associated with subsequent HCC development [6]. Qu et al. similarly observed progressively greater HCC risk with accumulation of the T1653, V1753, and T1762/A1764 mutations [7].

HCV RNA and Genotype

In a prospective community cohort of 925 anti-HCV-positive individuals, HCC risk increased according to HCV RNA status. Cumulative risk was 1.1% among HCV RNA-negative participants, 6.4% among those with low-level viremia, and 14.7% among those with high-level viremia; genotype 1 infection was associated with a cumulative HCC risk of 12.6% compared with 4.5% for non-genotype-1 infection [14]. Genotype 1b was also associated with HCC in earlier studies of cirrhotic populations. Silini et al. found genotype 1b to be more common among patients with HCC than among cirrhotic controls without HCC [15], while Bruno et al. reported genotype 1b in 19 of 22 patients who developed incident HCC and identified an OR of 6.14 [16].

HBV/HCV Coinfection and Occult HBV

Studies of patients with cirrhosis suggested that combined HBV and HCV infection may confer greater HCC risk than either infection alone. Benvegnù et al. observed the highest occurrence of HCC among patients with concurrent HBV and HCV infection [32]. Tsai et al. reported an HR of 6.41 (95% CI, 1.80-22.80) for HCC in coinfected patients, compared with HRs of 3.74 for anti-HCV positivity alone and 4.06 for HBsAg positivity alone [33]. Occult HBV infection was examined as a possible modifier of HCC risk in HCV-associated liver disease. Squadrito et al. reported that eight of nine incident HCC cases occurred among patients with occult HBV infection [34], while Ikeda et al. found that anti-HBc positivity was associated with greater HCC risk among patients with HCV-related cirrhosis, with an incidence rate ratio of 1.58 [35]. The evidence for occult HBV was less consistent and was based on fewer studies than that for overt HBV or HCV infection.

Comparative Histopathological Evidence

Muro et al. compared noncirrhotic HBV- and HCV-associated HCC and found broadly similar levels of fibrosis but significantly greater necroinflammatory activity in the background liver of patients with HCV-associated HCC [19]. Takazawa et al. reported that patients with HCV-related HCC were generally older and more frequently cirrhotic, whereas HBV-associated tumors tended to be larger and less differentiated [20]. Miyagawa et al. found greater synchronous multicentricity among patients with HCV-related HCC, together with older age and poorer underlying liver function [21]. These studies therefore showed a consistent tendency toward greater background inflammatory injury and advanced liver disease in HCV-associated HCC, while HBV-associated HCC was also observed in less fibrotic or noncirrhotic liver. Genotype-specific histopathological differences could not be assessed reliably because the included histopathological studies primarily compared HBV-associated and HCV-associated HCC rather than stratifying pathological findings according to individual viral genotypes. Although HCV genotype 1b was associated with increased HCC risk in some included studies [15,16], these studies did not provide sufficiently detailed genotype-stratified histopathological data to support a separate morphological comparison.

HBV Molecular Integration and Genomic Evidence

The included genomic studies consistently demonstrated HBV DNA integration within HCC tissue. Sung et al. reported integration in 86.4% of HBV-positive tumors compared with 30.7% of adjacent nontumorous liver and identified recurrent involvement of TERT, KMT2B/MLL4, and CCNE1 [9]. Jiang et al. identified 255 HBV integration sites and showed associations with gene disruption, viral-host fusion transcripts, copy-number changes, and clonal expansion [10]. Toh et al. demonstrated promoter-enriched integration, recurrent TERT involvement, and structural alterations involving the HBx region [11]. More recent sequencing studies identified integration-associated rearrangements, translocations, driver copy-number alterations, integrated HBV concatemers, MYC amplification, and cis-regulatory effects [12,13]. Across these independent studies, recurrent TERT involvement and structural genomic disruption were consistent findings, supporting a direct molecular contribution of HBV integration to hepatocarcinogenesis [9-13].

The genomic findings were complemented by virological evidence. HBeAg positivity was strongly associated with subsequent HCC [3], increasing HBV DNA showed a biological gradient of risk [4], moderate-to-high viral load remained associated with HCC in noncirrhotic chronic hepatitis B [8], and high-risk enhancer II and basal core promoter variants provided an additional viral genetic association with HCC [5-7].

HCV Molecular and Epigenetic Evidence

The included HCV molecular studies identified viral-sequence and epigenetic abnormalities associated with HCC, although the molecular evidence base was smaller than that available for HBV integration. Fishman et al. identified several HCV core-gene polymorphisms associated with advanced liver disease and HCC, although prospective confirmation was considered necessary [17]. In a small tumor-tissue study, Rüster et al. observed greater core-region sequence variability in HCC tissue than in adjacent nontumorous liver, including substitutions, premature stop codons, and frameshift deletions [18]. Hlady et al. provided evidence of persistent epigenomic changes associated with HCV infection; enhancer and chromatin alterations remained detectable after viral clearance and were demonstrated in human samples [22]. Together, these observations suggest that HCV-associated hepatocarcinogenesis is closely linked to persistent viral infection and progressive liver injury, with viral-sequence variation and durable epigenetic alterations representing additional molecular features.

Residual HCC Risk After HCV Eradication

Kondili et al. evaluated 2,064 patients with cirrhosis who achieved sustained virological response after direct-acting antiviral therapy. During follow-up, 119 patients developed de novo HCC, with a cumulative incidence of 1.90% at 12 months, 4.21% at 24 months, and 6.47% at 36 months [23]. These findings demonstrate that successful HCV eradication does not completely eliminate HCC risk in patients with established cirrhosis.

Risk of Bias in Included Studies

The large prospective studies evaluating HBV and HCV virological markers generally had the lowest methodological concern because exposure was assessed before HCC development, follow-up was substantial, outcome ascertainment was systematic, and important confounders were considered [3,4,8,14]. Nested case-control studies and smaller cohorts were judged to have some concerns, primarily because of residual confounding, selected populations, and limitations of historical viral assays [5-7,15,16,32-35]. Overall, the HBV genomic studies were technically valid and consistently demonstrated integration and genomic changes, but concerns included population representativeness, selection of surgical or tumor tissue, and difficulty distinguishing potentially causative integration events from passenger events [9-13]. The smallest tumor-based investigations, particularly Rüster et al. and Muro et al., were judged to have high concern because of small sample sizes and consequent limitations in precision and generalizability [18,19].

The study-level methodological appraisal, including judgments for selection, exposure or assay validity, comparator and confounding, outcome or molecular validity, and overall risk of bias, is presented in Table 2 and graphically summarized in Figure 2.

**Table 2:** Study-level risk-of-bias/methodological appraisal NA, not available

Study	Selection	Exposure/assay	Comparator/confounding	Outcome/molecular validity	Overall
Yang et al. 2002 [3]	Low	Low	Low	Low	Low concern
Chen et al. 2006 [4]	Low	Low	Low	Low	Low concern
Kao et al. 2003 [5]	Some	Low	Some	Low	Some concerns
Chou et al. 2008 [6]	Low	Low	Low	Low	Low concern
Qu et al. 2014 [7]	Some	Low	Some	Low	Some concerns
Kim et al. 2020 [8]	Low	Low	Low	Low	Low concern
Sung et al. 2012 [9]	Some	Low	Some/NA	Low	Some concerns
Jiang et al. 2012 [10]	Some	Low	Some/NA	Low	Some concerns
Toh et al. 2013 [11]	Some	Low	Some/NA	Low	Some concerns
Qian et al. 2024 [12]	Some	Low	Some/NA	Low	Some concerns
Wang et al. 2025 [13]	Some	Low	Some/NA	Low	Some concerns
Lee et al. 2010 [14]	Low	Low	Low	Low	Low concern
Silini et al. 1996 [15]	Some	Low	Some	Some	Some concerns
Bruno et al. 1997 [16]	Some	Low	Some	Low	Some concerns
Fishman et al. 2009 [17]	Some	Some	Some	Some	Some concerns
Rüster et al. 2001 [18]	High	Some	NA	Some	High concern
Muro et al. 1996 [19]	High	Low	Some	Some	High concern
Takazawa et al. 1996 [20]	Some	Low	Some	Low	Some concerns
Miyagawa et al. 1996 [21]	Some	Low	Some	Low	Some concerns
Hlady et al. 2022 [22]	Some	Low	Some/NA	Low	Some concerns
Kondili et al. 2023 [23]	Low	Low	Some	Low	Low to some concerns
Benvegnù et al. 1994 [32]	Some	Low	Some	Low	Some concerns
Tsai et al. 1997 [33]	Some	Low	Low	Low	Some concerns
Squadrito et al. 2006 [34]	Some	Some	Some	Low	Some concerns
Ikeda et al. 2007 [35]	Some	Low	Some	Low	Some concerns

**Figure 2:**
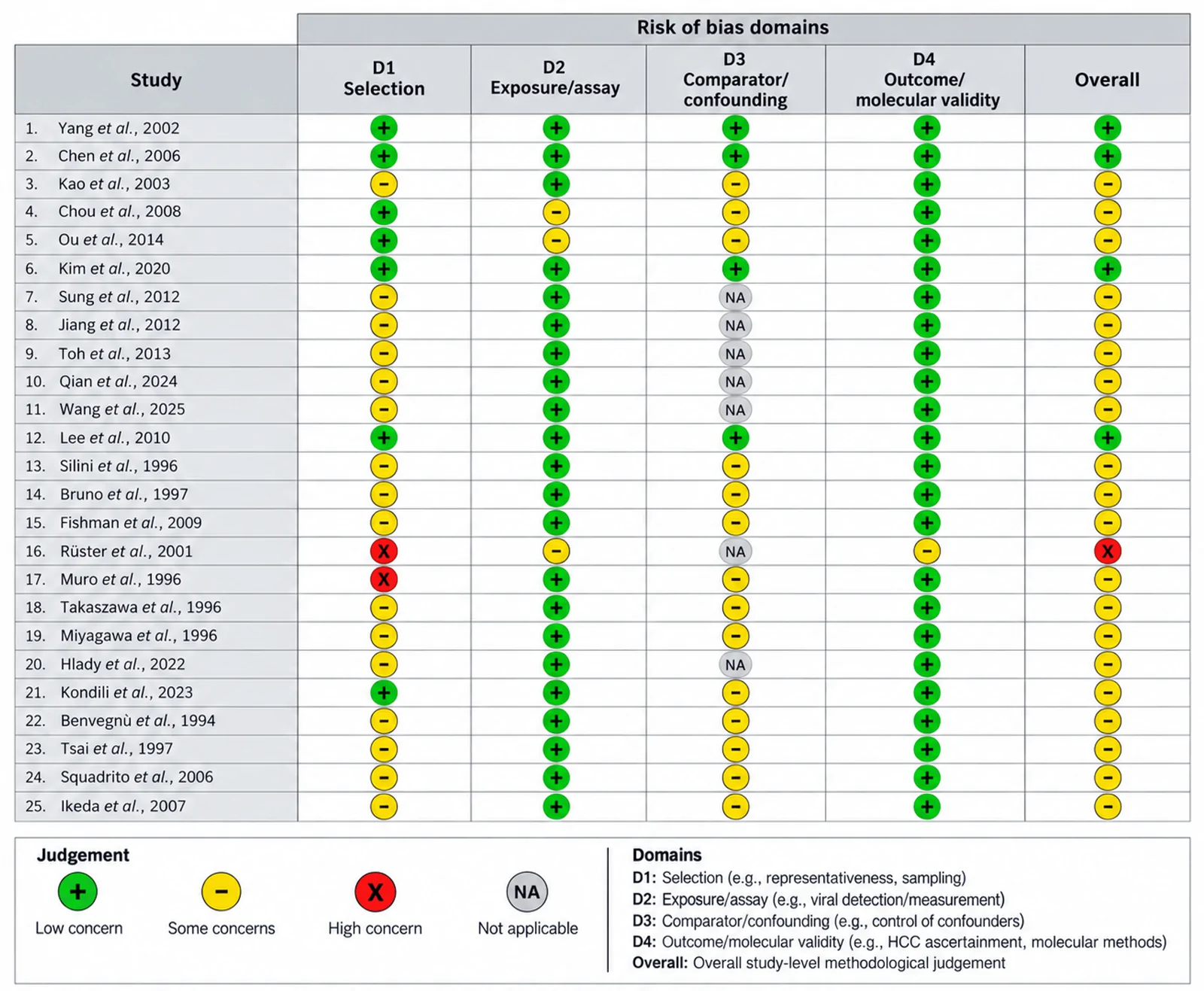
Graphical representation of the study-level risk-of-bias/methodological appraisal Green (+) indicates low concern, yellow (-) indicates some concerns, red (X) indicates high concern, and gray (NA) indicates not applicable. D1 = selection; D2 = exposure/assay; D3 = comparator/confounding; D4 = outcome/molecular validity; Overall = overall study-level methodological judgment. Assessments were based on study-design-specific domains informed by the JBI critical appraisal framework and the Q-Genie tool. The traffic-light plot was generated using robvis, an R package (R Foundation for Statistical Computing, Vienna, Austria) and a Shiny web application (Posit, Boston, MA) for visualizing risk-of-bias assessments in systematic reviews [36] JBI, Joanna Briggs Institute; Q-Genie, Quality of Genetic Studies; robvis, Risk-of-Bias VISualization

Certainty of Evidence

The certainty of evidence across the different synthesis domains is summarized in Table 3.

**Table 3:** Certainty of evidence by synthesis domain Certainty assessments were based on the included studies contributing to each synthesis domain: HBV serological/viral-load association [3,4,8]; HBV viral mutations [5-7]; HCV RNA/genotype association [14-16]; HBV/HCV coinfection and occult HBV [32-35]; comparative histopathology [19-21]; HBV integration/genomic evidence [9-13]; HCV molecular/epigenetic evidence [17,18,22]; and residual HCC risk after HCV SVR [23] HBV, hepatitis B virus; HCV, hepatitis C virus; HCC, hepatocellular carcinoma; SVR, sustained virological response

Evidence domain	Evidence base	Certainty	Reason for judgment
HBV serological/viral-load association	Large prospective cohorts with long follow-up	Moderate	Strong temporal sequence, large effects, and dose-response relationship; observational design limits certainty
HBV viral mutations	Nested/case-control molecular epidemiology	Low to moderate	Repeated high-risk variant associations and some temporal evidence, but smaller studies and residual confounding
HCV RNA/genotype association	Prospective cohorts and cirrhosis studies	Low to moderate	Dose-response for viremia and repeated genotype signal; heterogeneity by geography and treatment era
HBV/HCV coinfection and occult HBV	Prospective cohorts and occult-infection studies	Low	Direction generally supportive but limited by imprecision, assay heterogeneity, and inconsistent occult-HBV findings
Comparative histopathology	Small-to-moderate clinicopathological series	Low	Generally consistent direction, but selected surgical populations and small sample sizes
HBV integration/genomic evidence	Multiple independent tumor genomic studies	Moderate	Consistent integration enrichment and genomic convergence; selected tumor populations and non-random sampling
HCV molecular/epigenetic evidence	Small sequence studies and epigenomic study	Low	Biological plausibility and human molecular evidence, but relatively small evidence base and uncertainty regarding causal direction
Residual HCC risk after HCV SVR	Large treated cirrhosis cohort	Moderate	Direct longitudinal evidence with clinically relevant incidence; primarily applicable to patients with cirrhosis

Overall, the certainty of evidence was judged to be moderate for HBV serological/viral-load associations, HBV integration/genomic evidence, and residual HCC risk after HCV sustained virological response. Certainty was low to moderate for HBV viral mutations and HCV RNA/genotype associations, and low for HBV/HCV coinfection and occult HBV infection, comparative histopathological evidence, and HCV molecular/epigenetic evidence. These judgments reflected differences in study design, sample size, consistency, potential confounding, assay validity, and the directness of the available evidence.

Results of Sensitivity Analysis

The qualitative sensitivity analysis assigned minimal interpretive weight to the two small tissue studies judged to have high methodological concern [18,19]. The main conclusions remained unchanged: active HBV replication remained supported by large prospective studies, HBV integration remained supported by multiple independent genomic investigations, and HCV-associated HCC remained closely associated with persistent viral infection, advanced liver disease, and molecular alterations.

Reporting Bias

Formal assessment of small-study effects was not performed because no quantitative meta-analysis was undertaken. Using the qualitative ROB-ME-informed assessment described above, publication bias was considered particularly plausible for small exploratory molecular and tumor-tissue studies, in which positive viral variants, integration events, or genomic abnormalities may have been more likely to be reported than null findings. Selective outcome reporting could not be formally evaluated for many older observational and molecular studies because prospective protocols and prespecified analytical plans were uncommon. Larger prospective cohort studies were considered less susceptible to this form of bias than small exploratory tissue-based investigations.

Discussion

Principal Findings

This systematic review integrates epidemiological, histopathological, genomic, and molecular evidence from 25 primary human studies examining the relationship of HBV and HCV infection with HCC. Both viruses are strongly associated with HCC, but the relative contribution of direct viral mechanisms and chronic liver injury differs between them.

Prospective cohort studies provided the strongest epidemiological evidence for HBV. HBeAg positivity was associated with a marked increase in subsequent HCC risk [3], increasing serum HBV DNA showed a clear biological gradient [4], and this association remained evident in more recent non-cirrhotic cohorts [8]. Viral sequence variation, particularly basal core promoter and enhancer II variants, provided an additional layer of risk [5-7].

Genomic findings further support a direct carcinogenic component of HBV. Independent sequencing studies repeatedly identified HBV DNA integration in TERT and other cancer-associated loci, together with viral-host fusion transcripts, copy-number alterations, chromosomal rearrangements, and other structural genomic abnormalities [9-13]. These observations provide a plausible mechanism by which HBV-associated HCC may develop before advanced fibrosis or cirrhosis in some patients.

The pattern was different for HCV. Increasing HCV RNA levels were associated with greater HCC risk [14], and several earlier studies linked genotype 1b with increased risk in cirrhotic populations [15,16]. Histopathological studies placed chronic necroinflammation and advanced liver disease more prominently in the HCV carcinogenic pathway: HCV-associated background liver generally showed greater inflammatory activity and a stronger association with cirrhosis, while HBV-associated HCC was also observed in noncirrhotic liver [19-21].

The HCV molecular evidence was smaller and less uniform than the HBV integration literature. Core-gene sequence variation and tumor-associated viral-sequence heterogeneity were reported [17,18], while persistent enhancer and chromatin alterations after viral clearance supported durable molecular changes after active infection had resolved [22]. Continued HCC risk after sustained virological response in patients with cirrhosis further supports the importance of preexisting structural and molecular liver injury [23].

Biological Interpretation of HBV-Associated Hepatocarcinogenesis

HBV-related HCC appears to result from an interaction between chronic hepatic injury and virus-specific molecular mechanisms. The direct component is particularly important because HBV DNA integration can alter host-gene regulation, create viral-human fusion transcripts, promote chromosomal instability, and affect cancer-related loci such as TERT [9-13].

This interpretation is consistent with mechanistic literature describing persistence of nuclear cccDNA, prolonged viral-protein expression, HBx-mediated interference with tumor-suppressor pathways, altered proliferative signaling, oxidative stress, and chronic inflammatory injury [24,25]. Other mechanistic work has similarly emphasized the interaction between HBV integration, HBx activity, genomic instability, and host-cell signaling in hepatocarcinogenesis [37]. cccDNA persistence should be distinguished from viral DNA integration: cccDNA acts as a stable nuclear transcriptional reservoir, while integrated HBV DNA represents a distinct genomic event capable of modifying host chromosomal structure and gene regulation.

Taken together, the epidemiological and genomic findings support two interacting pathways in HBV-associated HCC: progressive inflammatory liver injury and a direct viral-genomic pathway involving HBV integration and oncogenic deregulation. This framework explains why cirrhosis substantially increases risk but is not an obligatory intermediate step in every HBV-associated HCC.

Figure 3 summarizes these interacting mechanisms, including persistent cccDNA, HBV DNA integration, HBx-related signaling, disruption of tumor-suppressor pathways, immune escape, genomic instability, angiogenesis, and chronic inflammatory injury.

**Figure 3:**
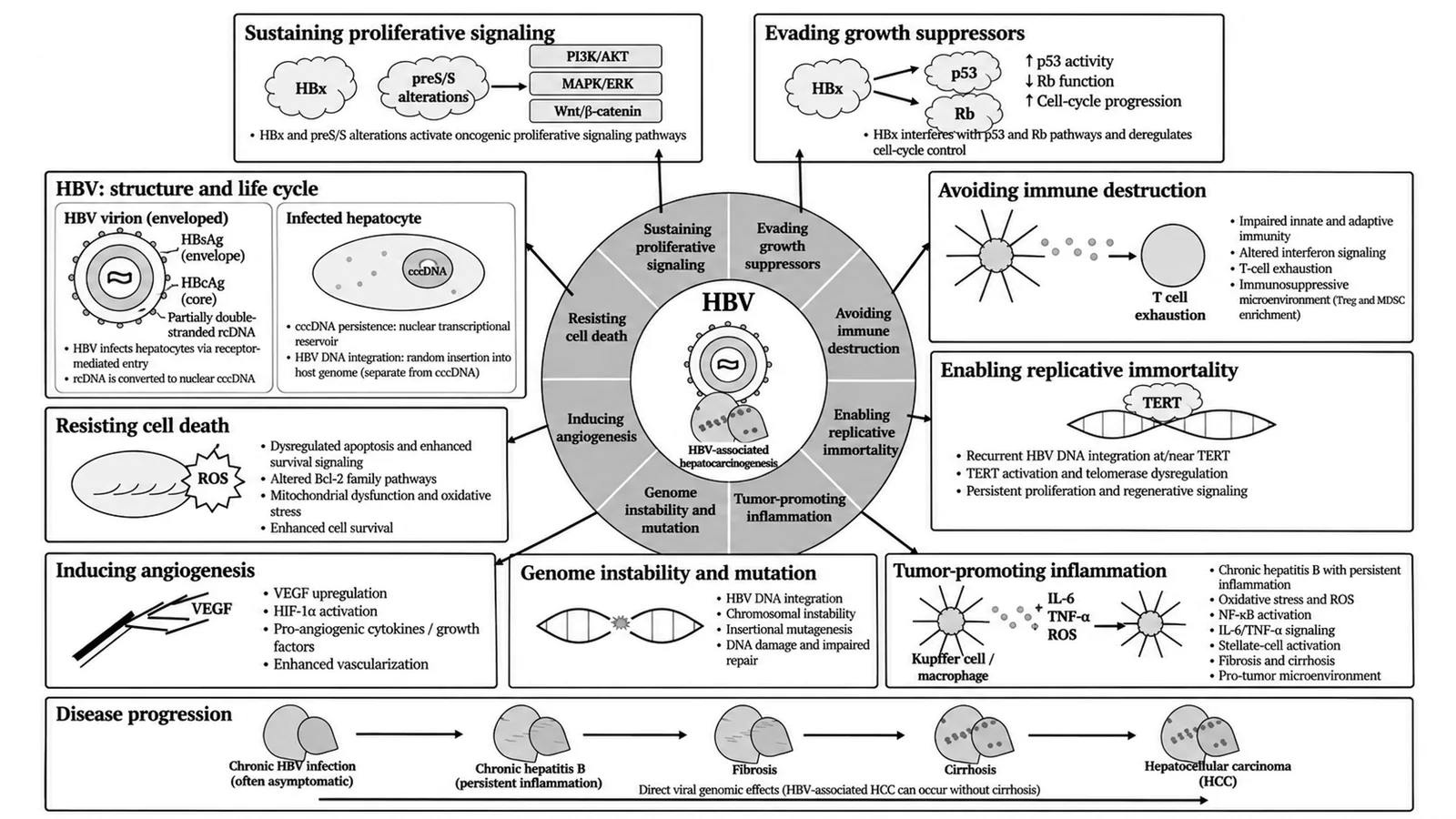
Selected hallmarks of cancer and molecular mechanisms of HBV-associated hepatocarcinogenesis The schematic summarizes major pathways through which persistent HBV infection contributes to hepatocarcinogenesis, including sustained proliferative signaling, evasion of growth suppression, immune dysregulation, resistance to cell death, angiogenesis, genomic instability, tumor-promoting inflammation, and the direct effects of HBV DNA integration. Persistent cccDNA supports ongoing viral transcription, while HBV integration can promote insertional mutagenesis and structural genomic disruption. Progressive inflammation and fibrosis may culminate in cirrhosis and HCC, although direct viral-genomic mechanisms can contribute before cirrhosis. HBV, hepatitis B virus; HCC, hepatocellular carcinoma; HBx, hepatitis B virus X protein; HBsAg, hepatitis B surface antigen; HBcAg, hepatitis B core antigen; rcDNA, relaxed circular DNA; cccDNA, covalently closed circular DNA; ROS, reactive oxygen species; VEGF, vascular endothelial growth factor; HIF-1α, hypoxia-inducible factor 1-alpha; TERT, telomerase reverse transcriptase; PI3K, phosphoinositide 3-kinase; AKT, protein kinase B; MAPK, mitogen-activated protein kinase; ERK, extracellular signal-regulated kinase; Rb, retinoblastoma protein; NF-κB, nuclear factor kappa B; IL-6, interleukin-6; TNF-α, tumor necrosis factor-alpha; Treg, regulatory T cell; MDSC, myeloid-derived suppressor cell Image credit: The image was created by the authors using Python 3.13.5 (Python Software Foundation, Wilmington, DE) and Matplotlib 3.10.8 (Matplotlib Development Team, United States)

Biological Interpretation of HCV-Associated Hepatocarcinogenesis

Unlike HBV, HCV does not normally integrate into host chromosomal DNA as part of its life cycle. Its dominant carcinogenic pathway is therefore more closely related to persistent infection and the cumulative effects of chronic inflammation, oxidative stress, repeated hepatocyte injury and regeneration, progressive fibrosis, and cirrhosis.

Mechanistic studies outside the primary synthesis have described effects of HCV proteins on cellular signaling, oxidative stress, mitochondrial and endoplasmic-reticulum function, apoptosis, and inflammatory pathways [25,38,39]. These mechanisms are biologically consistent with the direct human evidence but should be distinguished from the primary studies included in the review.

The included human molecular evidence showed HCV core-gene variation associated with advanced disease and HCC [17], tumor-associated sequence variability in a small tissue study [18], and persistent epigenomic alterations after viral clearance [22]. These findings indicate that HCV carcinogenesis is not attributable to fibrosis alone, although the human molecular evidence remains less extensive than the evidence for HBV integration.

Persistent epigenetic changes may also help explain why successful antiviral therapy does not immediately normalize cancer risk. In the PITER cohort, patients with cirrhosis remained at measurable risk of HCC after sustained virological response [23]. Once advanced fibrosis, cirrhosis, clonal alterations, or persistent molecular changes have developed, elimination of active viral replication may reduce carcinogenic pressure without completely reversing accumulated hepatic injury.

Figure 4 summarizes HCV-associated hepatocarcinogenesis as a predominantly inflammation-, fibrosis-, and molecular-regulatory process rather than an insertional oncogenic pathway.

**Figure 4:**
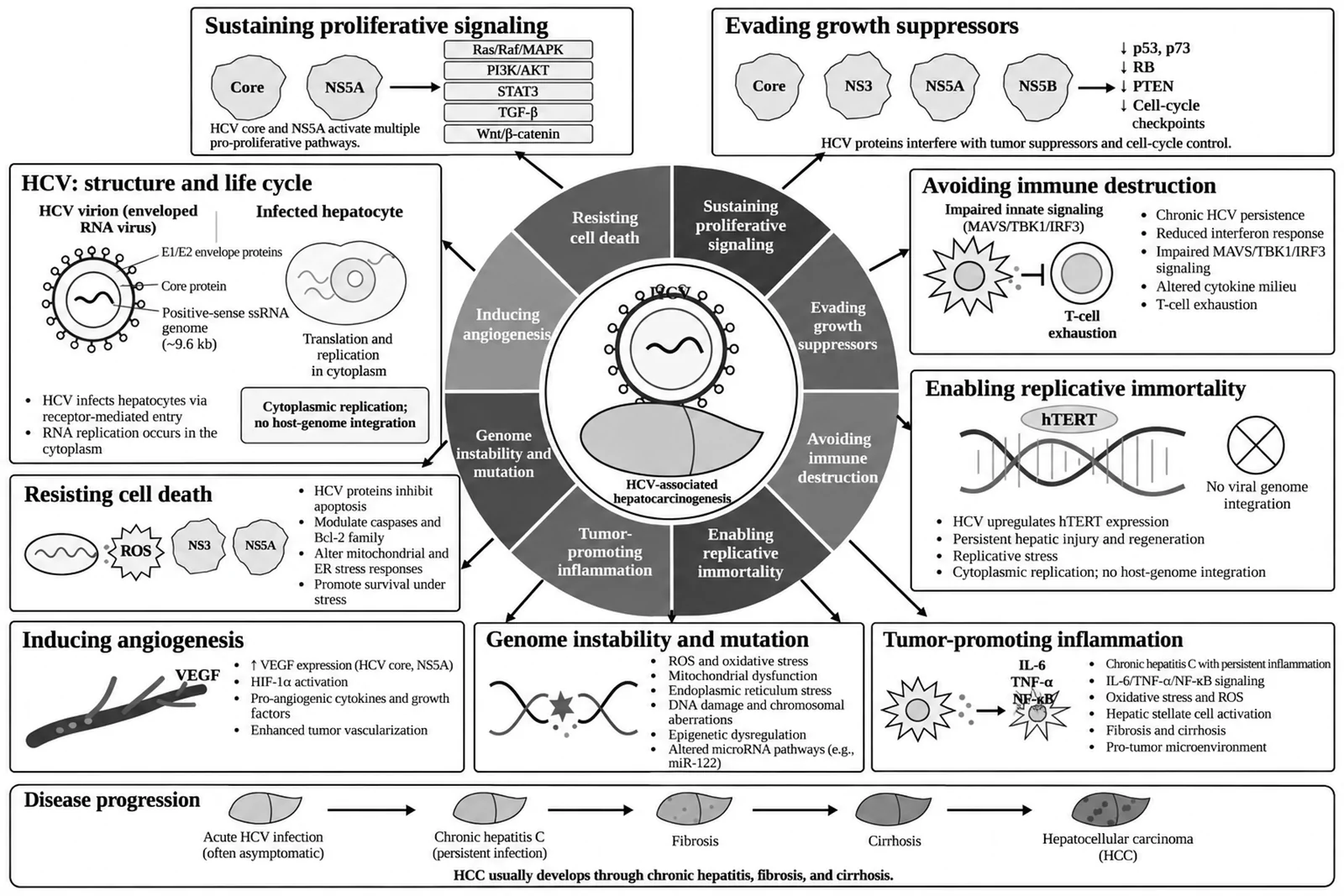
Selected hallmarks of cancer and molecular mechanisms of HCV-associated hepatocarcinogenesis The schematic summarizes major pathways through which persistent HCV infection contributes to hepatocarcinogenesis, including sustained proliferative signaling, evasion of growth suppression, immune dysregulation, resistance to cell death, angiogenesis, oxidative injury, genomic and epigenetic alterations, and tumor-promoting inflammation. Chronic hepatic injury promotes progression from chronic hepatitis C to fibrosis, cirrhosis, and HCC. HCV replicates predominantly in the cytoplasm and does not normally integrate into the host genome HCV, hepatitis C virus; HCC, hepatocellular carcinoma; ROS, reactive oxygen species; VEGF, vascular endothelial growth factor; HIF-1α, hypoxia-inducible factor 1-alpha; hTERT, human telomerase reverse transcriptase; NF-κB, nuclear factor kappa B; IL-6, interleukin-6; TNF-α, tumor necrosis factor-alpha; ER, endoplasmic reticulum Image credit: The image was created by the authors using Python 3.13.5 (Python Software Foundation, Wilmington, DE) and Matplotlib 3.10.8 (Matplotlib Development Team, United States)

Comparison of HBV- and HCV-Associated HCC

The distinction between HBV and HCV carcinogenesis is one of relative predominance rather than absolute separation. Chronic inflammation, oxidative injury, immune dysregulation, fibrosis, and regenerative proliferation contribute to HCC development in both infections. The most notable difference is the direct genomic contribution of HBV, in which viral DNA integration can participate in malignant transformation even before late-stage cirrhosis. In contrast, HCV-associated HCC more often develops as a complication of chronic inflammatory and fibrotic liver disease. These distinctions are supported by histopathological findings showing greater necroinflammatory activity and more advanced underlying liver disease in HCV-associated HCC [19-21].

The difference should not be overstated. HBV-associated HCC frequently occurs in patients with fibrosis or cirrhosis, while HCV may exert direct molecular effects through viral proteins, oxidative damage, sequence variation, and epigenetic alterations. The evidence therefore supports overlapping mechanisms in which direct virus-mediated effects and chronic hepatotoxic injury occupy different positions of relative importance.

HBV/HCV Coinfection and Occult HBV

Coinfection studies suggested that combined HBV and HCV infection may increase HCC risk beyond either infection alone. Benvegnù et al. reported the greatest HCC occurrence in coinfected patients [32], while Tsai et al. reported an HR of 6.41 for coinfection [33]. Evidence regarding occult HBV infection was less consistent. Squadrito et al. reported that most incident HCC cases in an HBsAg-negative chronic hepatitis cohort occurred in patients with occult HBV infection [34], whereas Ikeda et al. found anti-HBc positivity to be a risk marker for HCC among patients with HCV-related cirrhosis [35]. These findings are potentially important but should be interpreted in light of differences in occult-infection definitions, laboratory assays, and study populations.

Clinical Implications

The findings have several clinical implications. Cirrhosis should not be the sole consideration in HCC risk assessment in chronic HBV infection. Viral replication, HBeAg status, HBV DNA level, fibrosis stage, and established clinical risk factors should be considered when evaluating surveillance needs.

Effective antiviral therapy for HCV substantially reduces future liver-related risk, but HCC risk persists in patients with advanced fibrosis or cirrhosis. Post-SVR surveillance should therefore continue for patients who remain within established high-risk groups.

The molecular evidence also suggests future potential for viral-host genetic or epigenetic biomarkers of HCC risk. Circulating viral-human chimeric DNA, recurrent HBV integration patterns, and persistent HCV-associated epigenetic alterations are plausible candidates but require prospective validation before routine clinical use.

Antiviral treatment of chronic HBV infection is clinically important for reducing viral replication and subsequent liver-related complications; however, studies specifically evaluating the effect of HBV antiviral treatment on HCC incidence were not a predefined evidence domain of the present systematic review. The current synthesis therefore focused on HBV virological markers, viral mutations, integration, genomic alterations, and related mechanisms of hepatocarcinogenesis. The effect of individual nucleos(t)ide analog regimens on residual HCC risk warrants dedicated evaluation in treatment-focused systematic reviews.

Strengths

A major strength of this review is integrating evidence domains that are commonly considered separately. The synthesis included prospective virological studies, comparative histopathology, tumor sequencing, viral genetic studies, epigenomic investigations, and post-antiviral outcome data. It also brought together older longitudinal studies and newer whole-genome and long-read sequencing investigations, allowing well-established epidemiological associations to be interpreted alongside increasingly detailed molecular evidence. Separate consideration of HBV and HCV avoided treating viral hepatocarcinogenesis as a single biological process. Methodological appraisal, qualitative reporting-bias assessment, and certainty judgments were included to avoid giving equal interpretive weight to large prospective cohorts and small exploratory tissue studies.

Limitations of the Evidence

Several limitations affect interpretation of the included literature. Geographic distribution varied considerably, with much of the HBV evidence originating from East Asia and several HCV studies arising from European or Japanese populations. Viral genotype distributions, host characteristics, environmental exposures, and background treatment practices may therefore limit generalizability. Many earlier studies were conducted before widespread use of potent nucleos(t)ide analogs for HBV or direct-acting antivirals for HCV, so their risk estimates may not fully represent contemporary treated populations.

Several molecular and histopathological investigations involved relatively small, selected surgical or tumor-tissue samples. These studies are valuable for identifying biological mechanisms but are less suitable for estimating population-level effects. Associations identified in established tumor tissue also cannot always determine whether a molecular alteration initiated carcinogenesis or developed during tumor evolution.

Evidence regarding HCV genotype and occult HBV was less consistent than evidence for HBV DNA, HBeAg status, or HBV integration. Earlier genotype associations should be interpreted in the context of changes in HCV epidemiology and antiviral therapy. A previous meta-analysis reported an association between HCV genotype 1b and HCC, but heterogeneity across populations and studies remains important [40].

Limitations of the Review Process

This review relied primarily on PubMed/MEDLINE supplemented by citation searching. Relevant studies indexed exclusively in Embase, Scopus, Web of Science, or regional databases may therefore have been missed. Study screening, risk-of-bias assessment, and certainty assessment were performed by a single reviewer rather than independently by two reviewers, increasing the possibility of missed studies, extraction error, or subjective methodological judgments. Because the evidence varied substantially in population, viral exposure, histopathological measures, and sequencing methods, quantitative pooling was not undertaken; narrative synthesis was considered more appropriate than potentially misleading summary estimates.

Future Research

Prospective studies should investigate whether specific HBV integration patterns can be detected before clinical HCC and distinguish true oncogenic drivers from passenger events. Larger and more diverse populations, including long-read sequencing studies, are needed. Prospective validation of circulating HBV-human chimeric DNA and other integration-derived biomarkers is also required, particularly in patients with long-term viral suppression or HBsAg clearance.

For HCV, larger longitudinal studies should determine whether persistent epigenetic alterations after eradication have independent prognostic value and whether specific viral sequence variants identify patients with continued HCC susceptibility. Ultimately, integrated studies combining clinical fibrosis assessment, quantitative virology, histopathology, genomics, transcriptomics, and epigenomics may improve individualized estimates of residual HCC risk after antiviral therapy.

## Conclusions

HBV- and HCV-associated HCCs share a background of chronic hepatic injury but differ in the relative importance of their dominant carcinogenic mechanisms. HBV combines inflammation- and fibrosis-related injury with a distinct direct genomic component driven by viral DNA integration and related genomic instability, whereas HCV-associated carcinogenesis is more strongly linked to persistent inflammation, oxidative injury, progressive fibrosis, and cirrhosis, with additional viral-protein and epigenetic effects.

These differences have practical implications for surveillance after antiviral treatment. Suppression of HBV replication or eradication of HCV substantially reduces carcinogenic pressure but does not necessarily abolish HCC risk once advanced liver disease or persistent molecular injury is established. Long-term risk-based surveillance therefore remains important, particularly for patients with advanced fibrosis, cirrhosis, or other recognized HCC risk factors.

## Appendices

**Table 4:** PubMed/MEDLINE search strategy HBV, hepatitis B virus; HCV, hepatitis C virus; HCC, hepatocellular carcinoma; HBsAg, hepatitis B surface antigen; HBeAg, hepatitis B e antigen; HBx, hepatitis B virus X

Element	Search details
Database	PubMed/MEDLINE via PubMed
Coverage	Database inception through June 30, 2026
HCC concept	("Carcinoma, Hepatocellular"[Mesh] OR "hepatocellular carcinoma"[Title/Abstract] OR HCC[Title/Abstract])
HBV/HCV concept	(("Hepatitis B"[Mesh] OR "hepatitis B virus"[Title/Abstract] OR HBV[Title/Abstract] OR HBsAg[Title/Abstract] OR HBeAg[Title/Abstract]) OR ("Hepatitis C"[Mesh] OR "hepatitis C virus"[Title/Abstract] OR HCV[Title/Abstract] OR "HCV RNA"[Title/Abstract]))
Mechanism/outcome concept	(virolog*[Title/Abstract] OR histopatholog*[Title/Abstract] OR histolog*[Title/Abstract] OR patholog*[Title/Abstract] OR molecular[Title/Abstract] OR carcinogenesis[Title/Abstract] OR pathogenesis[Title/Abstract] OR "viral integration"[Title/Abstract] OR "HBV DNA integration"[Title/Abstract] OR HBx[Title/Abstract] OR "viral load"[Title/Abstract] OR genotype*[Title/Abstract] OR mutation*[Title/Abstract] OR fibrosis[Title/Abstract] OR cirrhosis[Title/Abstract] OR "oxidative stress"[Title/Abstract] OR epigenetic*[Title/Abstract])
Final Boolean combination	HCC concept AND HBV/HCV concept AND mechanism/outcome concept
Filters	No automatic article-type filter; eligibility determined during screening
Supplementary searching	Backward citation searching of included studies and highly relevant background articles
